# Supplementary material for: Pre-clinical evaluation of antiviral activity of nitazoxanide against SARS-CoV-2
Source: eBioMedicine. 2022 Jul 11;82:104148. doi: 10.1016/j.ebiom.2022.104148 (PMC9271885; doi:10.1016/j.ebiom.2022.104148)
Supplement: Supplementary file 2 [file mmc2.docx]

| Parameter | Population estimates ^a^  (%RSE) ^b^ | 95%CI^b^ | IIV ^a^ [%CV]  (%RSE) ^b^ | 95%CI ^b^ |
| --- | --- | --- | --- | --- |
| F | 1 (fixed) | - | - | - |
| CL/F (L/h) | 0.651 (11.9) | 0.504-0.866 | 40.9 (26.5) | 14.8-53.5 |
| V/F (L) | 0.128 (48.3) | 0.044-0.359 | - | - |
| Q/F (L/h) | 0.391 (73.5) | 0.134-1.373 | 55.7 (156) | 26.8-220 |
| VP/F (L) | 0.262 (32.5) | 0.120-0.426 | - | - |
| k_a_ (h^-1^) | 1.74 (56.2) | 0.941-5.945 | 99.6 (33.4) | 40.8-267 |
| σ | 0.151 | 0.088-0.204 | - | - |
