# Supplementary material for: Pre-clinical evaluation of antiviral activity of nitazoxanide against SARS-CoV-2
Source: eBioMedicine. 2022 Jul 11;82:104148. doi: 10.1016/j.ebiom.2022.104148 (PMC9271885; doi:10.1016/j.ebiom.2022.104148)
Supplement: Supplementary file 3 [file mmc3.docx]

|  | Time post-treatment | Plasma (µg/mL) | Lung (µg/g) | L/p ratio | Nasal turbinates (µg/mL) |
| --- | --- | --- | --- | --- | --- |
| Multiple Dose : 2.8mg/kg/day TID (at 3 dpi) | 12 hours | 0.01 £  (0.03µM) | 0.10 ; 0.11 $  (0.39 ; 0.41µM/g) | 1279.5 | 0.01 £  (0.06µM) |
