# Supplementary material for: Pre-clinical evaluation of antiviral activity of nitazoxanide against SARS-CoV-2
Source: eBioMedicine. 2022 Jul 11;82:104148. doi: 10.1016/j.ebiom.2022.104148 (PMC9271885; doi:10.1016/j.ebiom.2022.104148)
Supplement: Supplementary file 4 [file mmc4.docx]

| **Gene Target** | **Primer and probes sequences** | **Amplicon length** | **Reference** |
| --- | --- | --- | --- |
| Sars-CoV-2 RNA-dependent RNA polymerase | Fwd: 5'-GTGARATGGTCATGTGTGGCGG-3' Rev: 5'-CARATGTTAAASACACTATTAGCATA-3' Probe: 5'-FAM-CAGGTGGAACCTCATCAGGAGATGC-TAMRA-3' | 99pb | Detection of 2019 novel coronavirus (2019-nCoV) by real-time RT-PCR (Corman et al.) |
|  |  |  |  |
|  |  |  |  |
| Syrian hamster γ-actin | Fwd: 5'-ACAGAGAGAAGATGACGCAGATAATG-3' Rev: 5'-GCCTGAATGGCCACGTACA-3' Probe: 5'-FAM-TTGAAACCTTCAACACCCCAGCC-TAMRA-3' | 70pb | Duplex real-time reverse transcriptase PCR to determine cytokine mRNA expression in a hamster model of New World cutaneous leishmaniasis (Espitita et al.) |
|  |  |  |  |
|  |  |  |  |
| Bacteriophage MS2 | Fwd: 5'-CTCTGAGAGCGGCTCTATTGGT-3' Rev: 5'-GTTCCCTACAACGAGCCTAAATTC-3' Probe: 5'-VIC-TCAGACACGCGGTCCGCTATAACGA-TAMRA-3' | 100pb | RNA and DNA Bacteriophages as Molecular Diagnosis Controls in Clinical Virology: A Comprehensive Study of More than 45,000 Routine PCR Tests (Ninove et al.) |
|  |  |  |  |
|  |  |  |  |
