# Supplementary material for: Pre-clinical evaluation of antiviral activity of nitazoxanide against SARS-CoV-2
Source: eBioMedicine. 2022 Jul 11;82:104148. doi: 10.1016/j.ebiom.2022.104148 (PMC9271885; doi:10.1016/j.ebiom.2022.104148)
Supplement: Supplementary file 5 [file mmc5.docx]

| **Lesion** | **Description** | **Intensity** | **Score** |
| --- | --- | --- | --- |
| **Interstitial pneumonia** | 1 o 2 foci with 10-20 cells or small area with two-fold thickening of alveolar septa | Mild | 1 |
|  | 3 to 5 foci with 10-30 cells or widespread areas with two-fold thickening of alveolar septa | Moderate | 2 |
|  | 5 foci of 10-50 cells or widespread areas with two-fold or three-fold thickening of alveolar septa throughout the lung | Marked | 3 |
|  |  |  |  |
|  | 5 foci of 10-100 cells or widespread areas with three to fourfold-thickened alveolar septa throughout the lung | Severe | 4 |
| **Bronchitis** | 1 or 2 bronchi section(s) filled with rare necrotic/inflammatory cells or partially surrounded by scarce inflammatory cells | Mild | 1 |
|  | 3 to 5 bronchi filled with necrotic/inflammatory cells or partially surrounded by a few inflammatory cells | Moderate | 2 |
|  | 6 to 10 bronchi filled with necrotic/inflammatory cells, partially or sub-completely surrounded by inflammatory cells | Marked | 3 |
|  | Numerous bronchi filled with inflammatory or cellular debris or completely surrounded by numerous inflammatory cells | Severe | 4 |
| **Endothelitis, vasculitis** | Absent | - | 0 |
|  | Present | - | 1 |
| **Hemorrhagic necrosis** | Absent | - | 0 |
|  | Focal to multifocal | Mild to moderate | 1 |
|  | Coalescing to extensive necrosis | Severe | 2 |
