# Supplementary material for: Pre-clinical evaluation of antiviral activity of nitazoxanide against SARS-CoV-2
Source: eBioMedicine. 2022 Jul 11;82:104148. doi: 10.1016/j.ebiom.2022.104148 (PMC9271885; doi:10.1016/j.ebiom.2022.104148)
Supplement: Supplementary file 6 [file mmc6.docx]

| **Cumulative score** | **Grade** | **Bronchointerstitial pneumonia** |
| --- | --- | --- |
| 0 | 0 | Normal |
| 1-3 | 1 | Mild |
| 4-5 | 2 | Moderate |
| 6-8 | 3 | Marked |
| 9-10 | 4 | Severe |
