# Supplementary figures and images for: Pre-clinical evaluation of antiviral activity of nitazoxanide against SARS-CoV-2
Source: eBioMedicine. 2022 Jul 11;82:104148. doi: 10.1016/j.ebiom.2022.104148 (PMC9271885; doi:10.1016/j.ebiom.2022.104148)

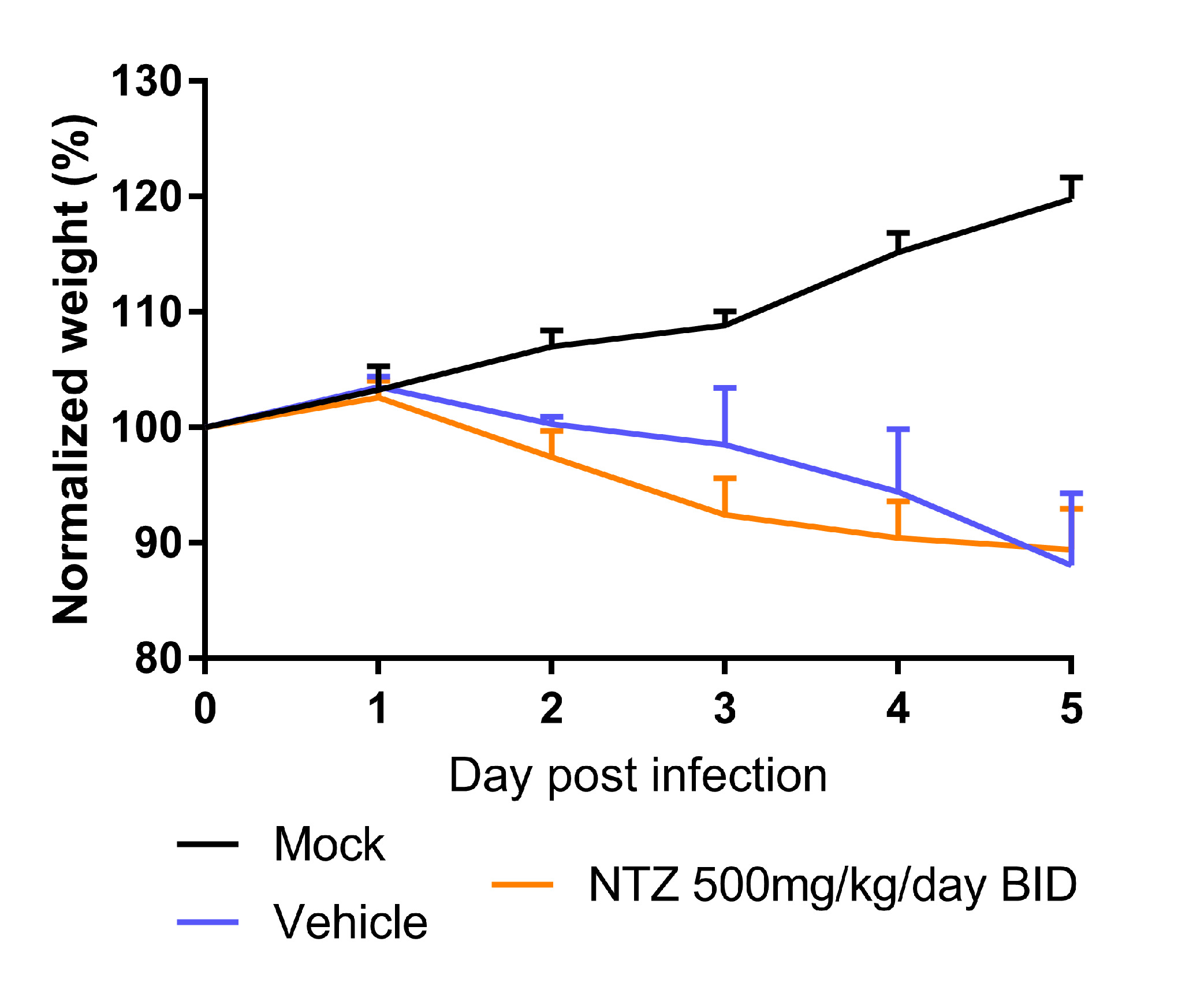

Supplement: Supplementary file 7 [file mmc7.jpg]

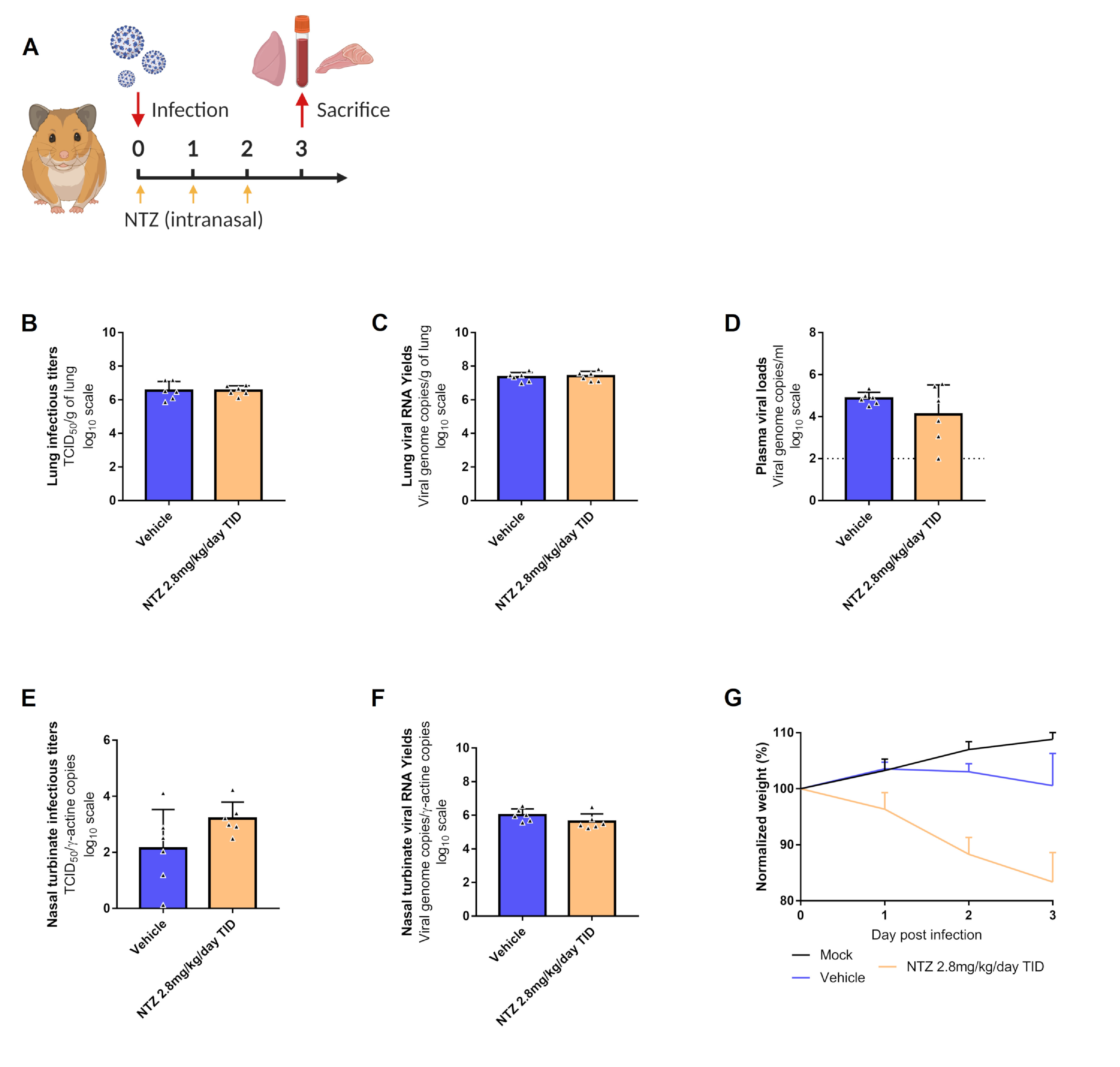

Supplement: Supplementary file 8 [file mmc8.jpg]

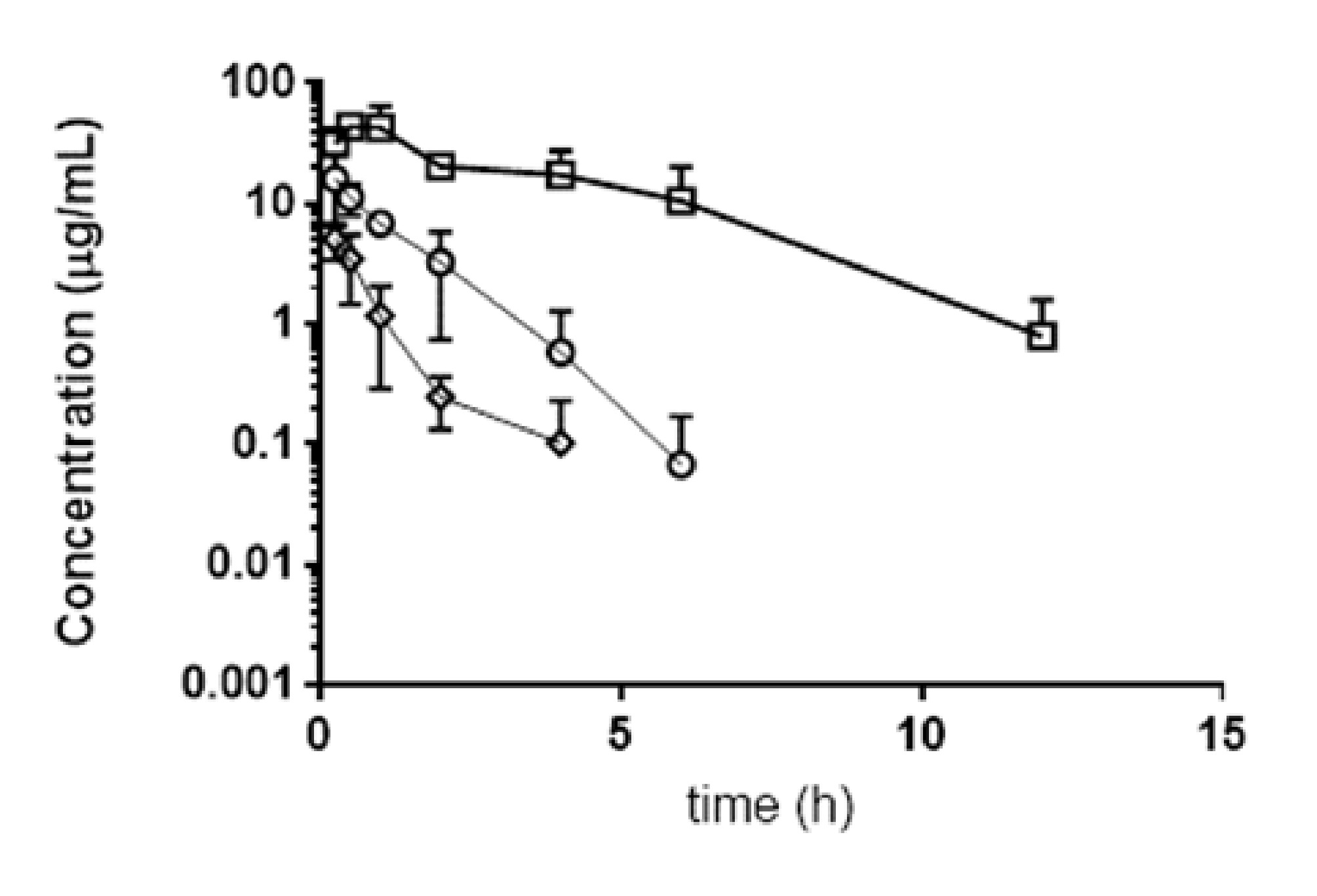

Supplement: Supplementary file 9 [file mmc9.jpg]

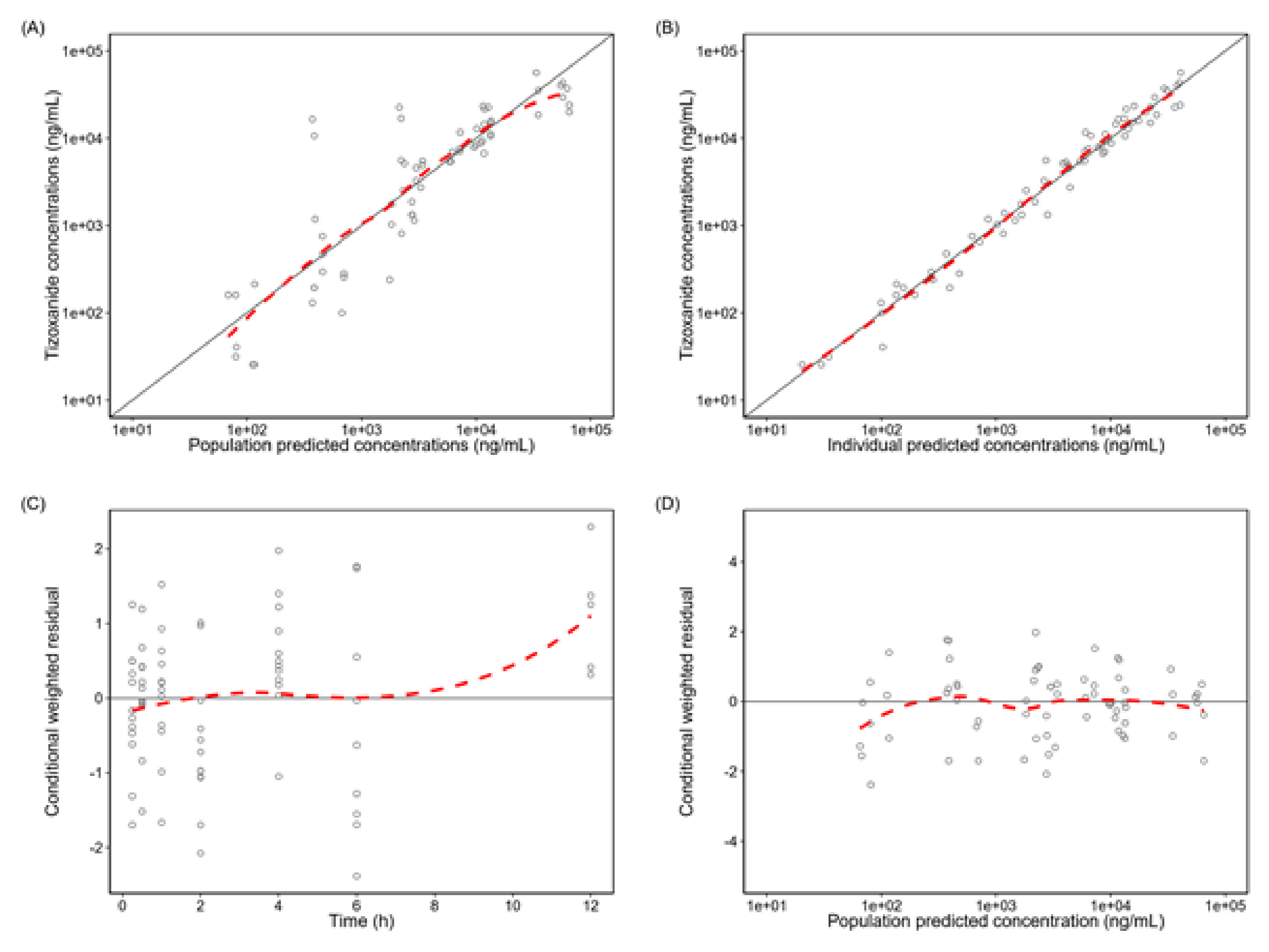

Supplement: Supplementary file 10 [file mmc10.jpg]
