# Supplementary material for: Pre-clinical evaluation of antiviral activity of nitazoxanide against SARS-CoV-2
Source: eBioMedicine. 2022 Jul 11;82:104148. doi: 10.1016/j.ebiom.2022.104148 (PMC9271885; doi:10.1016/j.ebiom.2022.104148)
Supplement: Supplementary file 11 [file mmc11.docx]

Supplementary Fig. 1: Clinical course of the disease (n=4 animals/group).

Normalized weight at day n was calculated as follows: % of initial weight of the animal at day n. Data represent mean ± SD (Details in Supplementary Data 2). Two-way ANOVA with Post-hoc Dunnett’s multiple comparisons test was performed.

Supplementary Fig. 2: Antiviral activity of intranasal treatment of NTZ in a hamster model.

Groups of 6 hamsters were intranasally infected with 10^4^ TCID_50_ of virus. **A** Experimental timeline. **B** Viral replication in lung based on infectious titers (measured using a TCID_50_ assay) expressed in TCID_50_/g of lung (n=6 animals/group). **C** Viral replication in lung based on viral RNA yields (measured using an RT-qPCR assay) expressed in viral genome copies/g of lung (n=6 animals/group). **D** Plasma viral loads (measured using an RT-qPCR assay) are expressed in viral genome copies/mL of plasma (the dotted line indicates the detection threshold of the assay) (n=6 animals/group). **E** Viral replication in nasal turbinates based on infectious titers (measured using a TCID_50_ assay) expressed in TCID_50_/copy of ɣ-actine gene (n=6 animals/group). **F** Viral replication in nasal turbinates based on viral RNA yields (measured using an RT-qPCR assay) expressed in viral genome copies/copy of ɣ-actine gene (n=6 animals/group). **G** Clinical course of the disease (n=6 animals/group). Normalized weight at day n was calculated as follows: % of initial weight of the animal at day n. Data represent mean ± SD (Details in Supplementary Data 2). Two-sided statistical analysis were performed using Shapiro–Wilk normality test, Fisher's exact test, Student t-test, Welch’s test and two-way ANOVA with Post-hoc Dunnett’s multiple comparisons test. ** symbols indicate that the average value for the group is significantly lower than that of the untreated group with a p-value ranging between 0.001-0.01 (Details in Supplementary Data 2 and 3).

Supplementary Fig. 3: Plasma concentration of TIZ following administration of NTZ to hamsters at doses of 485mg/kg (□), 98.1mg/kg (○) and 25.5mg/kg.

Three animals per dose group were included error bars represent the standard deviation.

Supplementary Fig. 4: Goodness-of-fit diagnostics of final nitazoxanide population pharmacokinetic model in hamster.

(**A**) Observed tizoxanide concentrations vs population predictions, (**B**) observed tizoxanide concentrations vs individually predicted concentrations, (**C**) conditionally weighted residual vs time, and (**D**) conditionally weighted residual vs population predictions. The open circles represent the observed tizoxanide concentrations. The solid black lines represent the line of identity and the dashed red lines represent a local polynomial regression fitting of all observations (i.e. trend line).

Supplementary Table 1: Plasma lung and nasal turbinates concentrations of TIZ after administration of multiple dose of NTZ.

PK realized after 3 days of nitazoxanide administered three times a day, at the end of the dosing interval (trough concentrations). Data represent individual values (Details in Supplementary Data 5). Symbols $ and £ represent respectively 4 or 5 values below the limit of quantification.

Supplementary Table 2: Population pharmacokinetic parameters from the final model of NTZ in hamster.

^a^ Population mean values, inter-individual variability (IIV) were estimated by NONMEM. The coefficient of variation (%CV) for IIV were calculated as $\text{100×}\sqrt{\exp\left( \text{estimate} \right)\text{-1}}$.

^b^ Relative standard error (%RSE) was calculated as $\text{100× (}\frac{\text{SD}}{\text{Mean value}}\text{)}$ from the non-parametric bootstrap results (n=1,000). The 95% confidence interval (95%CI) is presented as the 2.5 to 97.5 percentiles of bootstrap estimates.

(F : bioavailability ; CL/F : oral clearance ; V/F : volume of distribution of the central compartment ; Q/F : intercompartmental clearance ; VP/F : volume of distribution of peripheral compartment ; K_a_ : absorption rate constant ; σ : residual variability).

Supplementary Table 3: (RT)-qPCR systems.

Supplementary Table 4: Histopathological semi-quantitative lung inflammation scoring system

Supplementary Table 5: Histopathological lung inflammation semi-quantitative grading
